# Australian Aboriginal Children with Otitis Media Have Reduced Antibody Titers to Specific Nontypeable Haemophilus influenzae Vaccine Antigens

**DOI:** 10.1128/CVI.00556-16

**Published:** 2017-04-05

**Authors:** Ruth B. Thornton, Lea-Ann S. Kirkham, Karli J. Corscadden, Selma P. Wiertsema, Angela Fuery, B. Jan Jones, Harvey L. Coates, Shyan Vijayasekaran, Guicheng Zhang, Anthony Keil, Peter C. Richmond

**Affiliations:** a School of Paediatrics and Child Health, The University of Western Australia, Perth, Western Australia, Australia; b Telethon Kids Institute, The University of Western Australia, Subiaco, Western Australia, Australia; c Department of Otorhinolaryngology, Princess Margaret Hospital for Children, Subiaco, Western Australia, Australia; d School of Public Health, Curtin University, Perth, Western Australia, Australia; e PathWest Laboratory Medicine WA, Princess Margaret Hospital for Children, Subiaco, Western Australia, Australia; Vanderbilt University Medical Center

**Keywords:** Australian Aboriginal, indigenous, antibody, nontypeable Haemophilus influenzae, vaccines

## Abstract

Indigenous populations experience high rates of otitis media (OM), with increased chronicity and severity, compared to those experienced by their nonindigenous counterparts. Data on immune responses to otopathogenic bacteria in these high-risk populations are lacking. Nontypeable Haemophilus influenzae (NTHi) is the predominant otopathogen in Australia. No vaccines are currently licensed to target NTHi; however, protein D (PD) from NTHi is included as a carrier protein in the 10-valent pneumococcal polysaccharide conjugate vaccine (PHiD10-CV), and other promising protein vaccine candidates exist, including outer membrane protein 4 (P4) and protein 6 (P6). We measured the levels of serum and salivary IgA and IgG against PD, P4, and P6 in Aboriginal and non-Aboriginal children with chronic OM who were undergoing surgery and compared the levels with those in healthy non-Aboriginal children (controls). We found that Aboriginal cases had lower serum IgG titers to all NTHi proteins assessed, particularly PD. In contrast, serum IgA and salivary IgA and IgG titers to each of these 3 proteins were equivalent to or higher than those in both non-Aboriginal cases and healthy controls. While serum antibody levels increased with age in healthy controls, no changes in titers were observed with age in non-Aboriginal cases, and a trend toward decreasing titers with age was observed in Aboriginal cases. This suggests that decreased serum IgG responses to NTHi outer membrane proteins may contribute to the development of chronic and severe OM in Australian Aboriginal children and other indigenous populations. These data are important for understanding the potential benefits of PHiD10-CV implementation and the development of NTHi protein-based vaccines for indigenous populations.

## INTRODUCTION

Australian Aboriginal children experience an excessive burden of otitis media (OM) and associated hearing loss, which causes difficulties in language acquisition and learning and has long-term effects on their life course (1–3). While 10 to 20% of nonindigenous children develop chronic or recurrent OM, the condition is almost universal among Australian Aboriginal children by 12 months of age and persists to school age (4–6). OM is also endemic in other indigenous populations, such as Native American, Greenlandic and Alaskan Inuit, and Maori populations (7); however, the rates of OM reported for Australian Aboriginal children are the highest in the world (8).

OM occurs much earlier in Australian Aboriginal children than in their non-Aboriginal counterparts, often developing within weeks after birth (9). Disease onset at this time is associated with very early and dense nasopharyngeal colonization with the major otopathogens, i.e., nontypeable Haemophilus influenzae (NTHi), Streptococcus pneumoniae, and Moraxella catarrhalis (10, 11). NTHi is now the predominant pathogen isolated from the nasopharynx, middle ear effusion (MEE), and middle ear discharge of Australian Aboriginal and non-Aboriginal children with OM (12, 13). Specific antibody deficiencies may increase the susceptibility of children to both colonization and chronic infection with these otopathogens. Previously, we and others demonstrated that otitis-prone non-Aboriginal children under 3 years of age develop similar or higher antibody responses to conserved bacterial protein antigens, compared with their healthy, age-matched counterparts (14–19). We also recently demonstrated that Australian Aboriginal children with OM produce similar antibody titers in response to conserved proteins of S. pneumoniae (20). This finding is in contrast to other studies that observed impaired antigen-specific immune responses to proteins from NTHi and S. pneumoniae (21–25). Despite the almost universal nature of OM in Aboriginal communities and the predominance of NTHi disease, no studies have been conducted to assess specifically the naturally acquired NTHi-specific antibodies in Aboriginal children. These data are important to determine which surface-exposed proteins are immunogenic and/or appear to be protective, to help guide development of the best vaccine candidates for a broadly protective NTHi vaccine for Australian Aboriginal children.

Currently, a single vaccine containing an NTHi protein, i.e., protein D (PD), as a carrier protein in a 10-valent pneumococcal polysaccharide conjugate vaccine (PHiD10-CV) has been licensed. While licensure of PHiD10-CV was not for NTHi disease, in some trials this vaccine showed the potential to decrease NTHi-associated OM (26, 27), including in Australian Aboriginal children (26). Again, the data are conflicting, with several large randomized controlled trials not being able to demonstrate PHiD10-CV protection against acute OM (AOM) caused by NTHi (28–30). Interestingly, immunization with PHiD10-CV does not appear to significantly reduce nasopharyngeal carriage of NTHi (26–28, 31), suggesting that the effect of this vaccine is compartmental. This compartmental effect was also observed in animal models of disease, where reductions in NTHi infection of the middle ear cavity did not correlate with decreases in nasopharyngeal colonization (32). Data on naturally acquired anti-PD antibody titers in children with OM are again conflicting; our study in younger, otitis-prone, Australian non-Aboriginal children showed that they produced anti-PD antibody titers equivalent to those of non-otitis-prone children (14), whereas a study with “stringently defined” otitis-prone U.S. children observed deficiencies in anti-PD antibody titers (23). It has also been suggested that the protection afforded by PHiD10-CV against OM may be due not to induction of anti-PD antibodies but rather to the prevention of pneumococcal AOM and the initial damage that it may cause, which enables progression to complex OM (33). While the reduction of NTHi OM with PHiD10-CV immunization in some studies is promising, recent studies have indicated that the PD gene (*hpd*) is missing in a substantial subset of NTHi strains (34–36), which may enable vaccine evasion and expansion of such lineages in the community (33). Multivalent vaccines that target multiple strains of NTHi colonizing the nasopharynx and causing OM are required to significantly reduce the burden of NTHi OM.

Outer membrane protein 4 (P4) and protein 6 (P6) are essential for NTHi adhesion, colonization, and subsequent infection (37) and thus represent attractive vaccine candidates for NTHi-related mucosal infections such as OM. These antigens are crucial in the pathogenesis of NTHi and, in preclinical animal models (38–40), they elicit protective antibody responses (38, 40–43) and enhance the mucosal clearance of NTHi (39–42, 44). There are conflicting data regarding whether otitis-prone children have impaired immunity to P4 and P6 (14, 23, 25). As both P4 and P6 are highly conserved and promising candidates that may be included in a multiprotein vaccine, it is important to understand whether they are immunogenic and whether population differences exist, particularly among those at greatest risk of OM.

The aim of this study was to determine the naturally acquired mucosal and systemic antibody responses to the NTHi antigens PD, P4, and P6 in Australian Aboriginal and non-Aboriginal children with OM, compared to healthy children without chronic disease. Such information is important for understanding the potential benefits of PHiD10-CV implementation in indigenous populations and for developing future protein-based NTHi vaccines.

## RESULTS

### Study population.

A total of 183 children were recruited, including 36 healthy children, who were all non-Aboriginal, and 70 Aboriginal and 77 non-Aboriginal children undergoing surgery for OM. None of the healthy control children had any significant history of respiratory disease (including OM), 74% of the non-Aboriginal cases and 63% of the Aboriginal cases were undergoing surgery for ventilation tube insertion, and 21% of the non-Aboriginal cases and 31% of the Aboriginal cases were undergoing surgery for tympanoplasty (Table 1). The majority of non-Aboriginal cases were undergoing surgery because of a diagnosis of OM with effusion (OME) (79%) and/or recurrent AOM (rAOM) (47%), whereas 61% of the Aboriginal children had current diagnoses of OME and 34% chronic suppurative OM (CSOM) (Table 1). One non-Aboriginal case was also undergoing a mastoidectomy because of mastoiditis. One Aboriginal case and 2 non-Aboriginal cases were concomitantly diagnosed with cholesteatomas.

**TABLE 1:** Study population

Characteristic	Healthy children	Aboriginal cases	Non-Aboriginal cases	*P*
No.	36	70	77	NA
Age (mean [range]) (yr)	8.3 (1.6–14.4)	6.7 (1.7–12.7)	5.0 (1.1–13.6)	<0.01
Age group (no. [%])				
1–3 yr	7 (19)	13 (19)	28 (36)	<0.01
4–6 yr	7 (19)	24 (34)	29 (38)	
7–11 yr	13 (36)	28 (40)	18 (23)	
>11 yr	9 (25)	5 (7)	2 (3)	
Male (no. [%])	13 (36)	39 (56)	39 (51)	0.20
Had attended day care (no. [%])	23 (64)	28 (40)	43 (56)	0.29
History of CSOM (no. [%])	0 (0)	26 (37)	8 (10)	<0.01
Current surgery (no. [%])				
VTI^*a*^	NA^*e*^	44 (63)	57 (74)	0.20
Myringotomy	NA	4 (6)	4 (5)	0.35
Tympanoplasty^*b*^	NA	22 (31)	16 (21)	0.10
Adenoidectomy	NA	1 (1)	10 (13)	0.02
Principal diagnosis (no. [%])				
OME	NA	43 (61)	61 (79)	0.05
rAOM	NA	21 (30)	36 (47)	0.02
CSOM	NA	24 (34)	6 (8)	<0.01
Immunizations up to date (no. [%])	36 (100)	52 (74)	69 (90)	0.15
Had received Prevenar (%)^*c*^	33	14	19	0.09
Receiving antibiotics at enrollment (%)^*d*^	0	3	12	0.02

a VTI, ventilation tube insertion.

b Seven tympanoplasties were performed due to failure of tympanic membrane healing after ventilation tube insertion.

c Healthy controls, *n* = 36; Aboriginal cases, *n* = 56; non-Aboriginal cases, *n* = 73.

d Healthy controls, *n* = 36; Aboriginal cases, *n* = 65; non-Aboriginal cases, *n* = 75.

e NA, not applicable.

Confounding factors associated with differences in antibody levels between Aboriginal cases, non-Aboriginal cases, and healthy controls were assessed. Age had the greatest effect on salivary IgA antibody titers against P4, P6, and PD (*P* ≤ 0.05), while having no effect on serum IgA or IgG or salivary IgG titers. All antibody titers reported were adjusted for age. Previous day care attendance and gender were demonstrated to have no significant effects on antibody titers to any of the antigens tested (data not shown).

### Middle ear effusion cultures.

A total of 106 MEE specimens from 74 children were cultured; of those, 23 MEE specimens from 19 children were culture positive (22% of MEE specimens from 26% of the children). Five MEE specimens from 5 children (3 Aboriginal and 2 non-Aboriginal) were culture positive for H. influenzae, and MEE specimens from the left and right ears of 1 non-Aboriginal child were positive for S. pneumoniae. Five MEE specimens from 3 children (2 Aboriginal and 1 non-Aboriginal) were positive for M. catarrhalis, while 7 MEE specimens from 6 children (4 Aboriginal and 2 non-Aboriginal) were positive for Pseudomonas aeruginosa. Finally, 4 MEE specimens from 4 children (3 Aboriginal and 1 non-Aboriginal) were positive for Staphylococcus aureus.

### Systemic production of IgG in response to all 3 NTHi proteins was significantly lower in Aboriginal children with OM than in non-Aboriginal children with OM and healthy controls.

**FIG 1:**
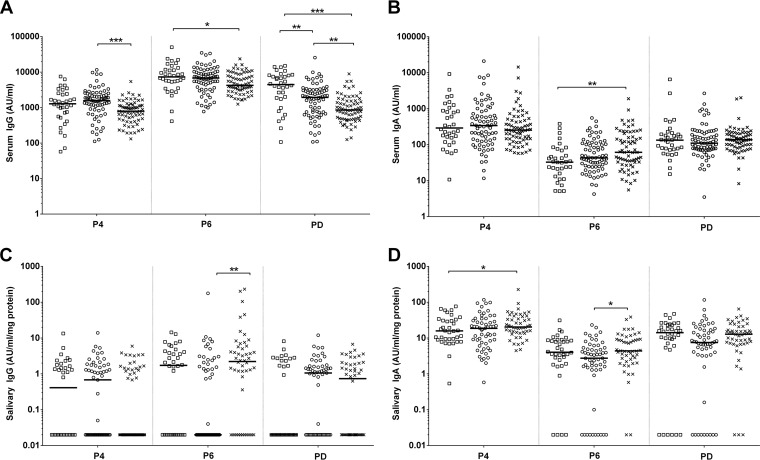
Comparison of serum IgG (A), serum IgA (B), salivary IgG (C), and salivary IgA (D) antibody titers between healthy non-Aboriginal children, Aboriginal children with otitis media, and non-Aboriginal children with otitis media. Levels of serum IgG and IgA antibodies and salivary IgG and IgA antibodies against NTHi proteins are presented for each individual child, with the horizontal bars depicting the medians. Statistical analyses were conducted on the geometric means of logarithmically transformed data, correcting for age. □, healthy controls; ○, non-Aboriginal children with OM; **×**, Aboriginal children with OM. P4, outer membrane protein 4; P6, outer membrane protein 6; PD, protein D. *, *P* ≤ 0.05; **, *P* ≤ 0.01; ***, *P* ≤ 0.001.

Aboriginal children with OM had significantly lower serum IgG titers to P4, P6, and PD than did non-Aboriginal children with OM or healthy controls (*P* ≤ 0.004) (Fig. 1A). Anti-PD serum IgG titers were significantly lower in both Aboriginal and non-Aboriginal children with OM than in healthy controls (*P* ≤ 0.003) and were also lower in Aboriginal children with OM than in non-Aboriginal children with OM (*P* = 0.002) (Fig. 1A). Anti-P4 serum IgG titers were significantly lower in Aboriginal children with OM than in non-Aboriginal children with OM (*P* ≤ 0.0001) and healthy controls (*P* ≤ 0.0001). Anti-P6 serum IgG titers were significantly lower in Aboriginal children with OM than in healthy controls (*P* = 0.042). Conversely anti-P6 serum IgA titers were significantly higher in Aboriginal children with OM than in healthy controls (*P* = 0.001) (Fig. 1B). Anti-P4 and anti-PD serum IgA titers were similar among the groups.

### Salivary antibody titers to all 3 NTHi proteins in Aboriginal children with OM were similar to or higher than those in non-Aboriginal children with OM and healthy controls.

Anti-P4 salivary IgA titers were significantly higher in Aboriginal children with OM (*P* = 0.050), but not non-Aboriginal children with OM, than in healthy controls (Fig. 1D). Both anti-P6 IgA and IgG titers were significantly higher in Aboriginal children with OM than in non-Aboriginal children with OM (*P* = 0.050 and *P* = 0.002, respectively) (Fig. 1C and D). There were no significant differences in anti-PD salivary IgG (Fig. 1C) or IgA (Fig. 1D) titers for any of the proteins between the groups; however, salivary anti-PD antibody titers tended to be higher in Aboriginal children with OM than in healthy controls.

The numbers of children who did not produce any detectable antigen-specific antibodies (i.e., nonresponders) were assessed among the groups. All children had detectable levels of serum IgG and IgA for all 3 NTHi proteins, while non-Aboriginal children with OM had more saliva samples below the limit of detection for anti-P6 IgA and IgG than did Aboriginal children with OM (*P* = 0.027 and *P* = 0.003, respectively). Healthy control children had more saliva samples below the limit of detection for anti-PD IgG than did non-Aboriginal children with OM (*P* = 0.017). No other differences in the numbers of nonresponders were observed between the groups.

### Antibody production in response to NTHi protein antigens occurs both mucosally and systemically.

Ratios of IgA to IgG antibody levels were compared for serum and saliva specimens, to determine whether antibodies in the saliva samples were mucosally produced or were present due to serum transudation. Highly significant differences (*P* ≤ 0.006) in the IgA/IgG ratios in serum and saliva samples were observed for antibodies to all 3 NTHi proteins, suggesting that IgG and IgA antibodies to these proteins are produced both systemically and mucosally. Spearman rho correlations were performed between serum and salivary IgA levels and serum and salivary IgG levels. Antibodies to all proteins had weak to moderate positive correlations (*r* = 0.221 to 0.465; *P* ≤ 0.009) for the entire cohort, in keeping with differential production at the mucosal and systemic levels.

### NTHi-antigen-specific antibody development during childhood differed between the populations, with a tendency to decrease with age in otitis-prone Aboriginal children.

Antigen-specific IgA and IgG production was correlated with the age of each child, to assess antibody development during childhood in the different groups (Tables 2 and 3). For healthy control children, moderate positive correlations between age and anti-P6 serum IgA titers (*r* = 0.544; *P* = 0.001), anti-PD serum IgA titers (*r* = 0.469; *P* = 0.005), and anti-PD serum IgG titers (*r* = 0.419; *P* = 0.014) were observed (Table 2). Weak positive correlations between age and anti-P6 salivary IgA titers (*r* = 0.393; *P* = 0.018) were also observed for healthy control children (Table 3). For non-Aboriginal children with OM, moderate positive correlations between age and anti-P6 salivary IgA titers (*r* = 0.582; *P* < 0.001) and anti-PD salivary IgA titers (*r* = 0.509; *P* < 0.001) were observed, and a weak positive correlation between age and anti-P4 IgA titers (*r* = 0.267; *P* = 0.045) was observed (Table 3). For Aboriginal children with OM, the only significant correlation between antibody titers and age that was observed was a weak negative correlation for anti-PD serum IgG titers (*r* = −0.346; *P* = 0.004) (Table 2). Anti-P4 and anti-P6 serum IgG titers were also negatively correlated with age for Aboriginal children with OM (Table 2); although those correlations did not reach significance, the findings do indicate that NTHi serum IgG titers do not appear to increase with age in Aboriginal children with OM.

**TABLE 2:** Correlations of serum IgA and IgG levels with age^*a*^

Antigen and group	Serum IgA		Serum IgG	
	Correlation coefficient (*r*)	*P*	Correlation coefficient (*r*)	*P*
P4				
Healthy controls	0.205	0.25	0.008	0.97
Non-Aboriginal children with OM	0.111	0.35	0.128	0.28
Aboriginal children with OM	−0.169	0.17	−0.231	0.06
P6				
Healthy controls	0.544	<0.01	0.039	0.83
Non-Aboriginal children with OM	0.014	0.91	0.112	0.35
Aboriginal children with OM	−0.127	0.30	−0.171	0.16
PD				
Healthy controls	0.469	<0.01	0.419	≤0.01
Non-Aboriginal children with OM	0.174	0.14	0.025	0.84
Aboriginal children with OM	0.183	0.13	−0.346	<0.01

a Weak correlations were defined as *r* = −0.3 to −0.1 or *r* = 0.1 to 0.3, moderate correlations as *r* = −0.5 to −0.3 or *r* = 0.3 to 0.5, and strong correlations as *r* = −1.0 to −0.5 or *r* = 0.5 to 1.0.

**TABLE 3:** Correlations of salivary IgA and IgG levels with age^*a*^

Antigen and group	Salivary IgA		Salivary IgG	
	Correlation coefficient (*r*)	*P*	Correlation coefficient (*r*)	*P*
P4				
Healthy controls	0.287	0.09	0.139	0.42
Non-Aboriginal children with OM	0.267	0.04	−0.007	0.96
Aboriginal children with OM	0.086	0.55	−0.058	0.69
P6				
Healthy controls	0.393	0.02	0.073	0.67
Non-Aboriginal children with OM	0.582	<0.01	0.195	0.15
Aboriginal children with OM	0.082	0.57	0.029	0.84
PD				
Healthy controls	0.279	0.10	0.110	0.52
Non-Aboriginal children with OM	0.509	<0.01	0.037	0.78
Aboriginal children with OM	0.132	0.36	0.126	0.38

a Weak correlations were defined as *r* = −0.3 to −0.1 or *r* = 0.1 to 0.3, moderate correlations as *r* = −0.5 to −0.3 or *r* = 0.3 to 0.5, and strong correlations as *r* = −1.0 to −0.5 or *r* = 0.5 to 1.0.

## DISCUSSION

Our data indicated that Australian Aboriginal children undergoing surgery for chronic or recurrent OM had impaired antigen-specific serum IgG antibody responses to NTHi antigens. This was particularly evident for PD, the carrier protein in PHiD10-CV, which is currently being assessed for its impact on OM in Australian Aboriginal children (the PREVIX-COMBO trial, registered at ClinicalTrials.gov under registration no. NCT01174849). Specifically, our data showed that Aboriginal children had approximately 25% of the serum IgG antibody responses to PD seen with healthy non-Aboriginal children, suggesting that either hyporesponsiveness or the development of immune tolerance to PD may occur in this high-risk population. This may contribute to the higher rates of NTHi infection and more severe disease observed in these children and has implications for vaccination with PHiD10-CV. These results do suggest that, when protection from NTHI OM is observed among PHiD10-CV-vaccinated children, it may not be only secondary to pneumococcal protection (33) but may be due to the development or boosting of anti-PD antibodies. Previous studies by our group did not identify aberrant immune responses to NTHi proteins in young non-Aboriginal children with rAOM (14); however, others demonstrated that otitis-prone children have poor responses to NTHi proteins following AOM (23, 25). NTHi-specific antibody deficiencies have also been described for adults with chronic obstructive pulmonary disease (COPD), diabetes mellitus, and chronic renal failure, who experience high rates of NTHi infection, compared to age-matched healthy controls (45). Antibody deficiency was particularly evident in adults with COPD, with 63% of patients having no detectable anti-PD serum IgG (45). These data suggest that populations at high risk of NTHi infection have impaired responses to NTHi antigens, particularly PD. A study by Pizzutto et al. demonstrated that both healthy children and children affected by chronic suppurative lung disease (CSLD), to whom Australian Aboriginal children disproportionately contribute, responded well to vaccination with PHiD-CV10; however, subanalyses of initial titers for healthy versus CSLD-affected children and Aboriginal versus non-Aboriginal children were not conducted (46). Our data suggest that these are important analyses to conduct and understand if we are to know what benefits PHiD-CV10 may elicit. Previous studies in which protection from NTHi-caused AOM by PHiD10-CV was not demonstrated (28–30) were conducted in populations with much lower rates of NTHi carriage, AOM, and NTHi-caused AOM, compared to high-risk indigenous populations such as Australian Aboriginal children (13). Furthermore, there are no data on antigen-specific antibody titers to PD in low-risk PHiD10-CV-vaccinated populations, to determine whether immunological deficiencies exist in these children that may be boosted by using the PHiD10-CV vaccine. Specific studies to determine whether PHiD10-CV immunization boosts anti-PD antibody titers in these high-risk populations and whether this contributes to protection from NTHi disease, as observed for OM in Aboriginal children immunized with PHiD10-CV (26), are warranted.

A recent study assessed the ontogeny of antibody responses to P6 and PD in children from birth to 6 years of age (47). The data indicated that children below the age of 1 month had the lowest antibody titers against P6 and PD; titers increased at 1 to 6 months, peaked at 7 months to 3 years, and then remained high at 4 to 6 years (47). Interestingly, in our study of children between 1 and 15 years of age, while an increase in PD IgG antibody titers with age was observed for healthy control children (Table 2), no increase was observed for P6 (or P4). No significant increases in antibody titers with age were observed for non-Aboriginal children with OM, and negative correlations between age and antibody titers for all proteins were observed for Aboriginal children, although the correlation was significant only for PD. This negative correlation suggests that there may be development of tolerance to these vaccine antigens that could affect both the initial susceptibility to disease and the implementation of vaccines in this population. A limitation to our study is that we did not collect nasopharyngeal swabs and therefore cannot comment on correlations between NTHi colonization and anti-PD antibody titers.

Among children, naturally acquired serum antibodies to PD are thought to be acquired in the first 2 to 3 years of life and appear to be bactericidal (22, 23, 48). High anti-PD IgG titers do not always correlate with neutralizing activity (49), however, and the functionality of the antibodies measured, rather than just titers alone, is likely to be important. A Finnish vaccination study suggested that it was the inhibitory function of the anti-PD antibodies, rather than titers, that was predictive of protection against OM (49); however, the enzymatic assay used has not been able to be reproduced by us or other groups.

Our study also showed that Australian Aboriginal children with OM had lower anti-P6 and anti-P4 serum IgG titers than did non-Aboriginal children with OM and healthy controls, despite having the same or higher serum IgA and salivary IgA and IgG titers to all proteins measured. These data suggest that serum IgG antibodies may be important in preventing OM or in promoting clearance of the underlying infection. Studies in animal models (rats, mice, and chinchillas) have shown that intranasal and subcutaneous vaccination with P6 and/or P4 elicits production of bactericidal antibodies that provide protection against the development of NTHi AOM and enhance mucosal clearance (38–42). Data for anti-P4 and anti-P6 antibodies in otitis-prone children are as conflicting as those for anti-PD antibodies (14, 23, 25), further suggesting that population-specific responses to NTHi need to be understood to enable development of an effective NTHi vaccine with broad global application, particularly for high-risk populations.

Our data indicate that poor serum IgG responses to NTHi proteins may contribute to the increased risk of severe or chronic OM observed in Australian Aboriginal children. This is important for understanding the potential benefits of PHiD10-CV implementation for indigenous populations and the ability of such children to respond to other potential NTHi vaccine candidates (i.e., P4 and P6). Whether vaccination with an NTHi protein, such as PD in PHiD10-CV, in infancy can boost antigen-specific antibody responses in Aboriginal Australian children and reduce the risk of developing OM has yet to be determined but is the subject of an ongoing clinical trial. Investigation into the causality of impaired NTHi protein responses in populations with high rates of NTHi exposure will contribute to our understanding of immune tolerance, which is essential for developing vaccines for these high-risk populations.

## MATERIALS AND METHODS

### Otitis media cases.

Children between 0 and 15 years of age were recruited at the time of admission for tympanoplasty or ventilation tube insertion, through public hospitals in urban and remote Western Australian hospitals (in Perth, Broome, and Derby), in 2003 to 2008. Written informed consent was obtained from the parents or guardians. Clinical data were collected using parental questionnaires and medical records.

Children were included if they met clinical criteria to undergo ventilation tube insertion for either OME or rAOM or tympanoplasty for CSOM. The diagnosis was recorded at the time of surgery. OME was defined as the presence of MEE, without symptoms or signs of suppurative infection (50), for longer than 3 months. rAOM was clinically defined, based on diagnoses by primary physicians and otorhinolaryngologists, as at least 3 AOM presentations within a 6-month period or 4 presentations within a 12-month period, between which clinical symptoms resolved (50). CSOM was defined as persistent discharge from the middle ear for more than 6 weeks through a perforation of the tympanic membrane (50); however, otorrhea was resolved prior to tympanoplasty. Approval for this study was obtained from the Princess Margaret Hospital for Children (ethics approval number 831EP), Armadale-Kelmscott Memorial Hospital, and Osborne Park Hospital ethics committees, the Western Australian Aboriginal Health Ethics Committee, and Aboriginal community-controlled health services in the Kimberley and Eastern Goldfields regions.

### Healthy controls.

Children between 2 and 15 years of age were recruited through the Vaccine Trials Group at the Princess Margaret Hospital for Children (Perth, Western Australia, Australia) between 2007 and 2009. Children were eligible if they were healthy and had no history of recurrent or chronic OM, no previous ear, nose, or throat surgery, no history of an obstructive sleep disorder, no history of chronic or recurrent sinusitis or rhinitis, and no history of pneumonia or chronic lung disease. Written informed consent was obtained from the parents or guardians. Clinical data were collected using parental questionnaires. Approval for this study was obtained from the Princess Margaret Hospital for Children ethics committee (ethics approval number 1385EP). Exclusion criteria for both cohorts included any chromosomal or craniofacial disorders, known immunodeficiency, or receipt of immunosuppressive therapy.

### Sample collection.

General anesthesia was used during the surgical procedures and, utilizing an operating microscope, an anteroinferior myringotomy incision was made. If MEE was present, then this was collected utilizing a 2-ml syringe with a blunt drawing-up needle. MEE samples were transported on ice within 4 h to the PathWest clinical microbiology laboratory at the Princess Margaret Hospital for Children, for bacteriological culture using standard culture techniques (20). Serum and saliva specimens were collected as described previously (20).

### Measurement of levels of IgG and IgA against NTHi proteins P4, P6, and PD.

The H. influenzae proteins P4 and P6 were expressed and purified as described previously (14); recombinant PD was provided by Wayne Thomas and Belinda Hales (46). Antibody titers were measured as described previously, using an in-house multiplex bead-based assay (14).

### Total protein assay.

To standardize salivary antibody levels across the cohort, total protein levels were measured using the Micro BCA (bicinchoninic acid) protein assay kit (Thermo Scientific), following the manufacturer's instructions, as described previously (20). Saliva antibody titers were standardized as arbitrary units (AU) per milligrams of total protein in the saliva (14).

### Statistical analyses.

Host and environmental risk factors were compared between Aboriginal and non-Aboriginal children with OM and healthy control children using Student's *t* tests for continuous variables (age) and Pearson's chi-square analyses (asymptotic significant 2-sided *P* values) for categorical variables (gender and day care attendance). Differences in frequencies of diagnoses and surgery, numbers of children in each age category, and numbers of samples below the assay detection limit for Aboriginal children versus non-Aboriginal children were assessed using chi-square analyses. Serum antibody titers were expressed as AU per milliliter against a reference human serum pool and are reported as adjusted antigen-specific geometric mean concentrations (GMCs), with 95% confidence intervals (CIs), correcting for age as a confounding variable. Adjusted GMCs were calculated by using logarithmically transformed, adjusted antibody concentrations. Salivary antibody titers were calculated as for serum titers and then were adjusted, to correct for interpatient variations in saliva production, by correcting the results to 1 mg/ml of total protein in the sample. Univariate analyses, using a general linear regression model and adjusting for age, were used to assess differences in anti-NTHi antibody levels between Aboriginal and non-Aboriginal children with OM and healthy control children. Paired-sample *t* tests were used to determine differences in IgA/IgG ratios in serum and saliva samples. The IBM SPSS Statistics 20 for Windows software package (IBM, Armonk, NY, USA) was used for all statistical analyses, and data were plotted using GraphPad Prism 6.0 (GraphPad Software Inc., La Jolla, CA, USA).
